# Synthetic molecular recognition nanosensor paint for microalbuminuria

**DOI:** 10.1038/s41467-019-11583-1

**Published:** 2019-08-09

**Authors:** Januka Budhathoki-Uprety, Janki Shah, Joshua A. Korsen, Alysandria E. Wayne, Thomas V. Galassi, Joseph R. Cohen, Jackson D. Harvey, Prakrit V. Jena, Lakshmi V. Ramanathan, Edgar A. Jaimes, Daniel A. Heller

**Affiliations:** 1 Memorial Sloan Kettering Cancer Center, New York, NY 10065 United States; 2 Department of Textile Engineering, Chemistry, and Science, North Carolina State University, Raleigh, NC 27695 United States; 3 Weill Cornell Medical College, New York, NY 10065 United States; 4 Washington University in St. Louis, St. Louis, MO 63130 United States

**Keywords:** Biosensors, Nanostructures, Blood proteins, Diagnostic markers

## Abstract

Microalbuminuria is an important clinical marker of several cardiovascular, metabolic, and other diseases such as diabetes, hypertension, atherosclerosis, and cancer. The accurate detection of microalbuminuria relies on albumin quantification in the urine, usually via an immunoturbidity assay; however, like many antibody-based assessments, this method may not be robust enough to function in global health applications, point-of-care assays, or wearable devices. Here, we develop an antibody-free approach using synthetic molecular recognition by constructing a polymer to mimic fatty acid binding to the albumin, informed by the albumin crystal structure. A single-walled carbon nanotube, encapsulated by the polymer, as the transduction element produces a hypsochromic (blue) shift in photoluminescence upon the binding of albumin in clinical urine samples. This complex, incorporated into an acrylic material, results in a nanosensor paint that enables the detection of microalbuminuria in patient samples and comprises a rapid point-of-care sensor robust enough to be deployed in resource-limited settings.

## Abstract (web summary)

Microalbuminuria, a clinical marker associated with cancer and hypertension, defined by low albumin levels in the urine, is normally detected by immunoassay. Herein, a nanosensor paint was developed using a polymer to mimic fatty acid binding to albumin, transduced by carbon nanotube fluorescence.

## Introduction

Albumin is the most abundant protein in the human body, constituting up to 60% of plasma and plays an essential role as a transporter of fatty acids^1^, biomolecules such as hormones^2^, and drugs^3^. Albumin is filtered in the kidneys and is only present in the urine at low concentrations in healthy individuals. A concentration in the urine above an established clinical threshold of ~30 mg/L represents a pathologic state. Diabetes^4^, cardiovascular diseases^5^, cancers^6^ and some therapeutic drugs^7^ trigger kidney injuries that may initiate long-term adverse effects^8^, leading to albuminuria—albumin protein leakage into the urine. Thus, albumin in the urine serves as a readily accessible, noninvasive biomarker of these diseases. Microalbuminuria describes lower concentrations, 30–300 mg/L of albumin in the urine^9^; persistent microalbuminuria may progress into macroalbuminuria (>300 mg/L urinary albumin), an indicator of severe kidney damage. Because conditions leading to microalbuminuria may often be controlled at early stages, the early detection and monitoring of microalbuminuria could greatly improve patient outcomes.

Currently, a high level of albumin in urine is most commonly detected by dipstick urinalysis^10^, but sensitivity is poor for microalbuminuria^11^. Other techniques (Supplementary Table 1) including chemical approaches using small molecule fluorescence, have been explored^12–14^; however, most are not robust enough for clinical laboratory assessments. The current clinical method for microalbuminuria detection is an immune-turbidimetry assay using polyclonal antibodies against human albumin^9^. Antibodies are highly sensitive, but they are expensive to produce, require refrigeration and careful handling, and are prone to degradation. Polyclonal antibodies are also subject to inherent batch-to-batch variations^15,16^. As such, they are poorly suited to limited-resource settings with underdeveloped infrastructure and point-of-care diagnosis. Thus, new methods are desirable to detect microalbuminuria in point-of-care and/or low-resource settings.

Synthetic molecular recognition denotes the development of molecular recognition elements that circumvent the need for natural recognition molecules such as antibodies. Nanoparticles can consist as templates for artificial molecular recognition^17–19^ serving as synthetic antibodies/immunosensors^20^. Many nanoparticles appear to bind to serum abundant proteins such as albumin non-specifically^21^. Fe–Pt nanoparticles^22^, quantum dots^23^, gold and silver nanoparticles^24^, and carbon nanotubes^25^ are some representative materials that bind to albumin. The binding ability of albumin to a wide range of materials provides both an opportunity for as well a challenge towards the development of an albumin-specific sensor.

Single-walled carbon nanotubes (SWCNTs) exhibit unique photophysical properties that enable the development of quantitative optical biosensors. These carbon nanotubes exhibit stable photoluminescence in the near-infrared region^26^, a suitable spectral window to measure responses in complex biofluid samples^27^ without interference from common metabolites fluorescent in the UV-visible range^28^. Carbon nanotube emission is sensitive to local dielectric, hydrophobicity, redox potential, and surface potential, enabling optical sensing^29^. Nanotube optical sensors have been employed to detect analytes such as oligonucleotides^30^, cancer antigens^31^, proteins^32^, neurotransmitters^33^, reactive oxygen species^34^, lipids^35,36^, and to measure strain^37^ in solid structures. We previously reported that photophysical properties and surface chemistry on SWCNTs can be modulated via encapsulation in helical polycarbodiimides^38^, polymers that enable unique control over nanotube surface chemistry, interactions with biomolecules and cells^39^, and facilitate subcellular targeting^40^.

Wearable and flexible sensors for biological and medical applications have recently garnered significant interest, and wearable sensors for the detection of heart rate and ECG have already reached the consumer electronics market^41^. Flexible electronics for the recording of cellular electronic activities^42^ and measurements of neurological activity have been under intense laboratory development for biological research applications, as well as eventual medical uses^43,44^. Sensor paints are under development for applications such as wound care^45^. Such materials could find applications in flexible and wearable sensors, which may transmit medical information to care providers in under-resourced settings^46^.

Herein, we investigated synthetic molecular recognition to modulate carbon nanotube photoluminescence response to albumin, which we then developed into a robust, rapid assay, in addition to a nanosensor paint for the detection of microalbuminuria. We designed and synthesized a suite of polycarbodiimide polymers to concomitantly encapsulate carbon nanotubes and interact with specific features of albumin based on the crystal structure. We found that nanotube-polymer complexes with a carboxylate-rich, hydrophobic polymer exhibited specific photoluminescence responses to albumin in the microalbuminuria range via both intensity enhancement and a hypsochromic shift (blue shift), potentially due to mimicry of the head group of albumin-binding fatty acids. The sensitivity of the response (up to 3 mg/L) is comparable to an immunoturbidimetry assay (1 mg/L)^47^, the most widely-used existing clinical method. The potential interferent proteins such as transferrin, γ-globulins, and degraded albumin elicited either negligible change or bathochromic instead of hypsochromic shifting of the nanotube emission, imparting selectivity in the testing conditions. We hypothesize that such selectivity is due to the carboxylate-functionalized polycarbodiimide acting as a molecular mimic of the head group of albumin-binding fatty acids, via both hydrophobic and Coulombic interactions, to provide synthetic molecular recognition of albumin. We found that the sensor functioned in clinical urine samples from human patients with microalbuminuria. To see if this sensor could be incorporated into a versatile form factor, we tested the functionality of the polymer-nanotube complexes in a scaffold that could be applied and dried onto surfaces as paint, and found that an acrylic mixture enabled retention of sensing ability. The resulting material is a free-standing optical sensor paint capable of measuring microalbuminuria in patient samples.

## Results

### Sensor development

**Fig. 1:**
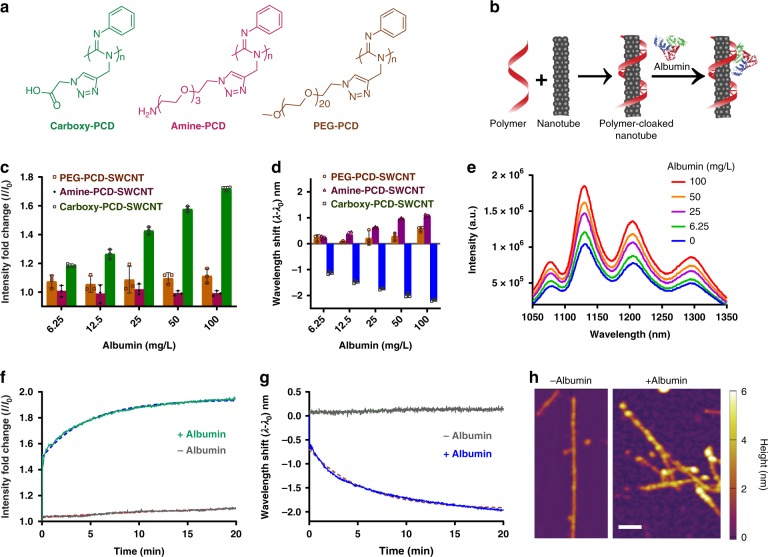
Optical nanosensor for albumin detection. **a** Schematic showing PCD structures. **b** PCD-SWCNT complex formation and its interaction with albumin protein. **c** Emission intensity response of the PCD-SWCNT complexes ((9, 4) chirality) to albumin. **d** Emission wavelength response of the PCD-SWCNT complexes to albumin ((9, 4) chirality). **e** Photoluminescence emission spectra of carboxy-PCD-SWCNT complexes upon addition of albumin (concentration increasing from bottom to top). **f** Response curve of emission intensity of carboxy-PCD-SWCNT complexes ((9, 4) chirality) to albumin. **g** Response curve of emission intensity of carboxy-PCD-SWCNT complexes to albumin ((9, 4) chirality). **h** Atomic fore microscopy height profile images of carboxy-PCD-SWCNT complexes and the same complexes upon exposing to albumin. Scale bar indicates 50 nm. Error bars represent standard deviation for *n* = 3 technical replicates

We synthesized polycarbodiimide-SWCNT (PCD-SWCNT) complexes to screen for specific and selective interactions with albumin. We synthesized polycarbodiimides polymers incorporating both phenyl rings to elicit non-covalent π–π interactions to the graphitic sidewall of the nanotube^38^ and diverse functional groups (Fig. 1a, Supplementary Fig. 1) to interact with analytes. Among them, we synthesized a hydrophobic carboxylated chain to mimic fatty acids, which are known to bind to albumin^48^. We also synthesized polymers with amine and polyethylene glycol (PEG) groups that could interact via Coulombic and hydrophobic interactions, respectively. The polymers and polymer-nanotube complexes (Fig. 1b) were synthesized and characterized as reported previously^38–40^. The optical properties of the polymer-nanotube complexes were confirmed by UV-VIS-NIR absorbance and NIR photoluminescence measurements as reported^38,39^. The various PCD-SWCNTs were exposed to 6.25–100 mg/L albumin, and their near-infrared photoluminescence spectra (900–1400 nm) were measured under 730 nm laser excitation. We focused on the response to the (9, 4) nanotube chirality due to its optical resonance at 730 nm and emission near 1120 nm, where there is minimal absorbance due to water or tissues, resulting in a high signal intensity from a relatively low concentration of nanotubes^36^. The response to albumin was a monotonic, dose-dependent emission intensity enhancement and wavelength shift towards shorter wavelengths (blue shift/hypsochromic shift) only for the carboxy-PCD-SWCNT sample (Fig. 1c–d). We observed a consistent blue-shift response from all observed photoluminescent nanotube species (Fig. 1e, Supplementary Figs. 2 and 3). The PEG-PCD-SWCNTs showed a negligible response to albumin, while the amine-PCD-SWCNTs responded via slight quenching of emission intensity and a small degree of emission red-shifting/bathochromic shifting (Fig. 1c, d). We attribute this response from amine-PCD-SWCNTs to accumulation of electrostatic charge on the nanotube surface and, potentially, agglomeration. The electrostatic attraction between the positively charged amine-PCD-SWCNTs and negatively charged albumin likely induced a bathochromic shift in the nanotube emission^49^. The electrostatic attraction between macromolecules may also prompt nanotube-nanotube agglomeration, which is known to cause photoluminescence quenching and red-shifting of nanotube emission via exciton dispersion^50^.

Upon identifying carboxy-PCD-SWCNTs as a potential sensor (henceforth referred to as the nanosensor) for albumin, we assessed the response kinetics. The results from the measurements showed that both the emission intensity (Fig. 1f) and blue shift (Fig. 1g) approached a maximum by 20 min with apparently pseudo first-order kinetics. The rate of intensity change was similar to that of the wavelength change with rate constants of 0.2140 min^−1^ and 0.1845 min^−1^, respectively.

We investigated the topology of the albumin-exposed nanosensor using atomic force microscopy (AFM). Height measurements on carboxy-PCD-SWCNT complexes/nanosensors showed that our preparation yielded singly-dispersed nanotubes with an average diameter of ~1–2 nm and median length of ~178 nm similar to previous findings^39^. The nanosensors were then incubated with albumin for 30 min at room temperature, excess albumin was removed via filtration, and the sample was deposited onto freshly cleaved mica, rinsed and dried before imaging via AFM under dry conditions. The images showed a resulting height increase of the nanosensors up to 4 nm (Fig. 1h, right panel, Supplementary Fig. 7), which closely resembles the size of albumin^24,51^, suggesting a monolayer deposition of albumin on the nanotube surface.

### Sensor response with other proteins

**Fig. 2:**
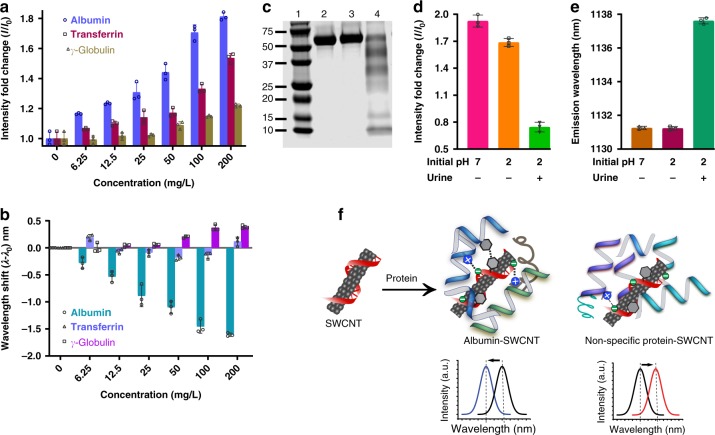
Sensor response to interferents, and sensor mechanism. **a** Emission intensity response of the nanosensor to various proteins. **b** Emission wavelength response of the nanosensor to protein interferents. **c** SDS-PAGE gel where: lane 2 contains albumin at pH 7 incubated at room temperature for 30 min; lane 3 contains albumin incubated at pH 2 in PBS at room temperature for 30 min followed by neutralization; lane 4 contains albumin incubated in urine at pH 2 for 30 min followed by neutralization. **d** Emission intensity response of the nanosensor to intact albumin (bars 1–2, corresponding to lanes 2–3, respectively, in panel **c**) and degraded albumin (bar 3, corresponding to lane 4 in panel **c**). **e** Emission wavelength response of the nanosensor to intact albumin (bars 1–2, corresponding to lanes 2–3 in panel **c**) and degraded albumin (bar 3, corresponding to lane 4 in panel C). **f** A proposed model of albumin interaction with the carboxy-PCD-SWCNT nanosensor. Data in panels **a**, **b** and **d**, **e** present the (9, 4) nanotube chirality. Error bars represent standard deviation for *n* = 3 technical replicates

We investigated whether the nanosensor could discriminate albumin from other proteins that could also be present in urine. Potentially interfering proteins were selected, including transferrin, which can leak into urine under abnormal kidney conditions^52^, and γ-globulin, indicative of glomerular damage^53^. We interrogated the nanosensors with transferrin and γ-globulin, resulting in significant, but attenuated intensity enhancement, as compared to albumin (Fig. 2a). Importantly, there was only a small change in emission wavelength (<0.5 nm), that became evident at higher doses (≥50 mg/L) of γ-globulin (Fig. 2b). Albumin derived from bovine, mouse, and human plasma all elicited consistent responses from the nanosensor (Supplementary Fig. 4A, B).

We hypothesized that if the binding pockets of albumin are disrupted by protein degradation, the sensor response could deviate from that observed with intact albumin and limit selectivity. Following a literature protocol^54^, we degraded albumin in urine under transient pH changes to low pH (incubated for 30 min in urine at low pH), as confirmed by SDS-PAGE analysis, Fig. 2c, and compared the sensor response in native and degraded albumin. In the case of degraded albumin, the sensor did not recapitulate the response. Degraded albumin attenuated the nanotube intensity (Fig. 2d), and elicited a bathochromic response, instead of a hypsochromic response (Fig. 2e). This is potentially consistent with loss of ligand binding capacity of proteins on degradation, wherein non-specific interactions of the degraded albumin may have caused the bathochromic response due to electrostatic interactions of the protein with the nanotube sidewall^40,49^.

We propose the following model for sensor interaction with albumin that results in blue-shifted emission (Fig. 2f) based on our experimental observations and known mechanisms of fatty acid binding to albumin^55^. Albumin presents high-affinity binding sites for fatty acids^56^. The crystal structure of fatty acid-bound albumin shows the presence of one or more basic amino acids (lysine/arginine/histidine) in hydrophobic pockets, which form salt bridges with the carboxylic acid on the fatty acid^57^ and aromatic amino acids^55^, which also exhibit affinity for the fatty acid^58,59^. We surmised that the structural similarities of the carboxylic acid-functionalized polycarbodiimide and the fatty acid, including the carboxylic acid and hydrophobic linker, on the polymer, could substitute for the fatty acid to promote the specific interactions, including salt bridge, hydrogen bonding, and hydrophobic interactions, between the fatty acid carboxylic head group^58,59^ and albumin. The presence of aromatic groups along the nanotube surface likely prompt π–π interactions with aromatic amino acid residues located in the binding pockets, further stabilizing the binding of albumin and displacing water from the nanotube surface.

The nanotube emission response behavior, upon interaction with albumin, is somewhat complex. The hypsochromic emission response is caused by the known solvatochromic behavior of nanotube emission^60^, caused here by displacement of water from the surface and interaction with hydrophobic residues on the protein. The intensity increase is likely similarly caused by the displacement of water from the nanotube surface, but is potentially affected most by the change in the redox environment upon removal of water^61^. The intensity change is thus faster than the hypsochromic response, and potentially exhibits two-component kinetics, as the curve does not fit precisely to a pseudo first-order model. The first component is likely due to adsorption resulting in rapid surface water displacement, followed by a slower conformational change of the protein on the sensor, which results in an increased hydrophobic environment on the nanotube surface. The intensity, more sensitive to the redox environment, changes most quickly, while the wavelength change is more strictly solvatochromic, responding most strongly to the hydrophobic interaction with the protein. The hydrophobic pockets are generally less accessible in soluble proteins and may interact with the nanotube only after conformation changes. In the case of sensor interaction with degraded albumin or other proteins, transferrin and γ-globulin, the response is mediated by mild water displacement, leading to an intensity increase, and a small degree of electrostatic interactions between the proteins and the nanotube surface, leading to a small bathochromic response^49^.

**Fig. 3:**
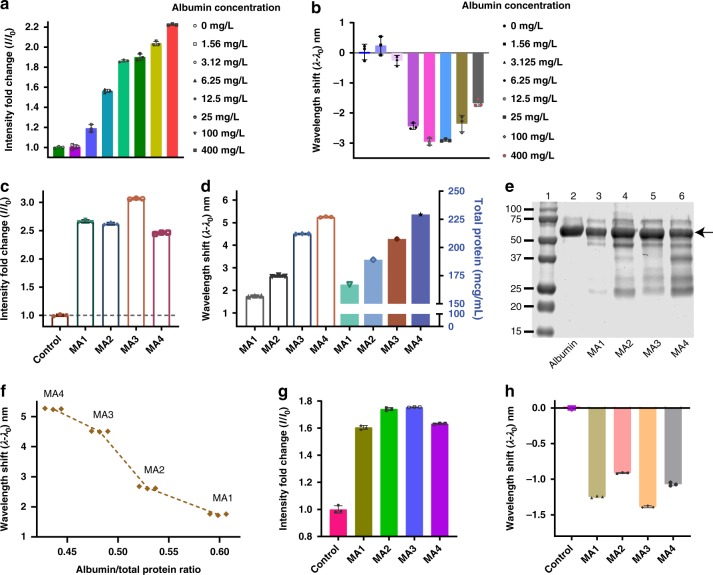
Sensor response in healthy and microalbuminuria patient samples. **a** Nanosensor emission intensity response to albumin spiked into human urine. **b** Emission wavelength response of the nanosensor to albumin in human urine. **c** Emission intensity response of the nanosensor to microalbuminuria patient samples. **d** Emission wavelength response of the nanosensor to microalbuminuria patient samples (left) and total protein determined via turbidimetry assay (right). **e** SDS-PAGE gel showing protein bands on microalbuminuria patient urine samples: (lane 1) molecular weight marker, (lane 2) albumin control, (lanes 3–6) microalbuminuria urine samples; arrow indicates albumin band. **f** Wavelength shift response from the nanosensor to total protein ratio in microalbuminuria urine samples. **g** Emission intensity response of the nanosensor to microalbuminuria urine samples after removal of interfering proteins. **h** Wavelength response of the nanosensor to microalbuminuria urine samples after removal of interfering proteins. The data presented in Fig 3a–h show the response of the (9,4) nanotube chirality. Error bars represent standard deviation for *n* = 3 technical replicates

We next tested our sensor in human whole urine samples. Urine from a healthy donor was collected and the quality of the urine assessed using a commercial dipstick urinalysis strip (Siemens Healthcare Diagnostics Inc.) to ensure absence of measurable proteins. Various concentrations of albumin, corresponding to microalbuminuria, were spiked into the urine. The sensor exhibited a response to the spiked albumin via enhanced emission intensity and a blue-shifted emission wavelength (Fig. 3a, b). In the whole urine sample, the blue shift exhibited a monotonic increase with albumin dose, while several high concentrations exhibited a maximum followed by a partially reversed response. This phenomenon was more prominent in the response of certain nanotube chiralities (Supplementary Fig. 3) and some conditions more than others, such as human urine (Fig. 3b), most likely due to molecular crowding. Such high concentration effects are common in macromolecular assays such as single-step immunological assays, including immunoturbidimetric assays^62^ and are generally corrected by dilution^63^.

We tested the nanosensor in human urine samples collected from patients with microalbuminuria. Waste human urine specimens collected with IRB approval were used at MSKCC. The nanosensor responded via intensity enhancement (Fig. 3c), similar to that previously observed in the albumin-spiked urine and in albumin in buffer conditions. However, notably, the emission wavelength exhibited a bathochromic response/red shift in the microalbuminuria samples (Fig. 3d). We investigated further via SDS-PAGE analysis of the urine samples, which showed a significant presence of several different proteins, including albumin plus other lower molecular weight proteins (Fig. 3e, panel 3–6). We then measured total protein in these samples via a standard clinical turbidimetry method^64^. The total protein measurement correlated with the nanosensor red-shifting response (Fig. 3d). On further analysis, we found that the ratio of albumin/total protein (Supplementary Table 2) strongly correlated with the degree of emission red-shifting (Fig. 3f). Small molecular weight proteins present in the microalbuminuria samples likely behaved in a similar manner as degraded albumin, investigated above, resulting in red-shifting behavior. Since the sensor responded to these samples with a net red shift, we conclude that the red shift from other interfering proteins competed with the blue-shifting response from albumin.

We investigated the de-convolution of the sensor response to albumin from the response to other proteins in the patient samples. We attempted to remove small molecular weight proteins via filtration through a 50 kD molecular weight cutoff membrane. On subsequent interrogation of the sensor with the filtered patient samples, the intensity was enhanced (Fig. 3g). The emission wavelength exhibited a distinct blue shift, however (Fig. 3h), similar to the response to albumin in buffer and spiked samples. We conclude that the removal of small molecular weight proteins from patient samples via filtration recapitulated the hypsochromic response of the sensor due to the removal of proteins that prompted electrostatic-induced bathochromic shifting behavior of the nanotubes.

### Paint-immobilized nanosensor

**Fig. 4:**
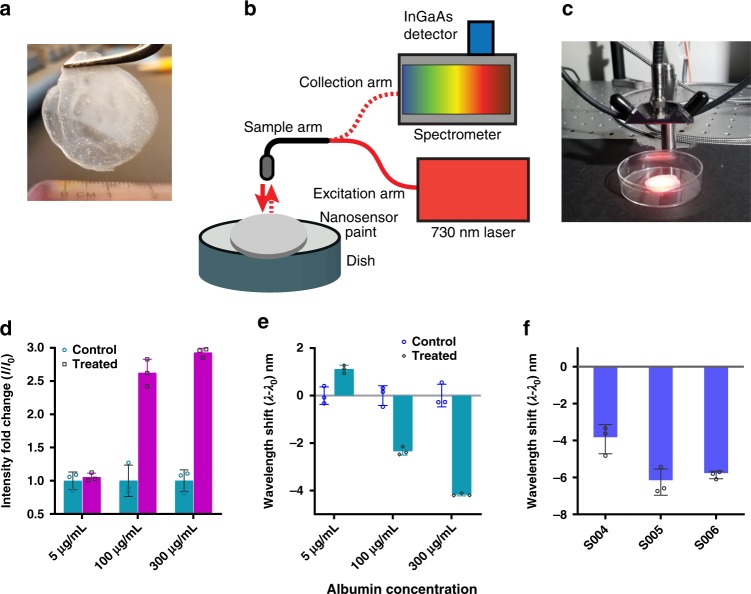
Albumin detection using free-standing nanosensor paint. **a** Nanotube blended with acrylic-based paint forming a free-standing film. **b** Schematic showing probe-based system used to excite nanotubes and collect NIR emission. **c** Photograph showing data acquisition using the probe-based system. **d** Emission intensity response of the nanosensor paint to albumin. **e** Emission wavelength response of the nanosensor paint to albumin. **f** Nanosensor paint response from microalbuminuria patient’s urine samples (S004, S005, and S006). The data in panels **d**–**f** show the response of the (9, 4) nanotube chirality. Error bars represent standard deviation for *n* = 3 technical replicates

We investigated the incorporation of the nanosensor into a form-factor that can be applied to surfaces. We blended the aqueous suspension of our nanosensor with a commercial varnish and casted it into a dry film (Fig. 4a). Albumin was applied on the surface of the film and the response was monitored using a custom-built fiber optic probe system (Fig. 4b, c) using a 730 nm continuous wave laser source and spectrometer/NIR array detector, previously described^31,61^. The paint-immobilized nanosensors exhibited emission enhancement (Fig. 4d) and a blue shift (Fig. 4e) in response to albumin (Supplementary Fig. 5). We then tested the nanosensor-embedded paint against urine samples from patients with microalbuminuria (Supplementary Table 3), after filtration using the 50 kD MWCO filters, described above. The sensors responded to these samples via a hypsochromic shift, largely as expected (Fig. 4f, Supplementary Fig. 6). The sensor-embedded paint exhibited some variations in center wavelength values, of ±1.12nm, and intensity differences of up to 2× (Supplementary Fig. 6). Therefore, the reference emission was collected individually for each measurement, corresponding to a location within the paint material. However, the relative changes in sensor response to albumin remained unaffected by the location. Interestingly, the paint-immobilized sensor exhibited higher magnitudes of intensity changes and wavelength shifts upon exposure to albumin. Although the exact mechanism for this observation remains to be elucidated, the improved signal might have resulted from combination of a number of factors such as a decrease in hydrophilic (water) content around nanotubes, and/or the acrylate paint, which contains carboxy moieties, may have facilitated favorable molecular interactions that improved protein binding to the paint/sensor material.

In conclusion, we investigated a synthetic molecular recognition optical nanosensor for the quantitative measurement of albumin in patients with microalbuminuria. The nanosensor was comprised of photoluminescent single-walled carbon nanotubes cloaked with a carboxylate-functionalized polycarbodiimide polymer–materials that afford the relative ease of production, storage, handling, stability, and robustness as compared to antibodies used in current methods. The rapid sensor response under ambient conditions in minimally processed urine samples suggests that this antibody-free detection may facilitate diagnosis in point-of-care and resource-limited settings.

The work investigated synthetic molecular recognition via polymer synthesis informed by protein structure. We surmise that binding and specificity of the polymer structure that conferred a unique optical response of the nanotube to albumin is mediated by electrostatic and hydrophobic interactions between the carboxylic acid functional groups on the polymer surface with basic amino acid groups in the binding pocket, in addition to hydrophobic/aromatic amino acid affinity to the hydrophobic polymer/nanotube structure.

The hypsochromic emission response of the sensor to albumin was caused by the known solvatochromic behavior of nanotube emission via displacement of water from the surface and interaction with hydrophobic residues on the protein. The intensity, more sensitive to the redox environment, likely changed quickly because of the rapid displacement of water upon binding of the albumin to the sensor, while the wavelength change was more strictly solvatochromic, responding most strongly to the hydrophobic interaction with the protein hydrophobic pockets, which are generally less accessible and may interact with the nanotube only after conformation changes. We found that the antibody-free detection of albumin exhibited a similar sensitivity compared to clinical immunoturbidimetry assays.

We also found that the nanosensor, integrated into a portable, free-standing paint, allows protein detection in patient urine samples. This “nanosensor paint” potentially opens new opportunities for engineering of point-of-care and wearable devices for clinical and field uses. The finding warrants further developmental steps towards these applications.

## Methods

### Materials

Reagents and solvents were purchased from Sigma–Aldrich, Milwaukee, WI, Acros Organics, and Fisher Scientific, Fair Lawn, NJ, and used as received. Anhydrous toluene and anhydrous and inhibitor-free diethyl ether were used in catalyst synthesis and purification. Anhydrous and inhibitor-free tetrahydrofuran was used for ‘click chemistry’. Bovine Serum albumin, mouse serum albumin, and human serum albumin were purchased from Sigma–Aldrich.

### Preparation of polymer-nanotube complexes

To prepare aqueous suspensions of polycarbodiimide single-walled carbon nanotube complexes, HiPco SWCNTs (Unidym, Lot # R1901, 1.5 mg) were added to an aqueous suspension of polycarbodiimide polymer functionalized with either primary amine side chains (amine-PCD, 5 mg/mL, pH ~3, total volume 1 mL) or functionalized with carboxylic acid side chains (carboxy-PCD, 5 mg/mL, pH ~ 9, total volume 1 mL). The polymer synthesis and characterization has been described in Supplementary Note 1. The heterogenous mixtures were sonicated at low temperature by using a probe-tip sonicator for 20 min (750 W, 20 kHz, 40% Amplitude, SONICS VibraCell). The solution was kept at low temperature using a CoolRack M30 PF (BioCision), precooled at −20 °C. The resulted dark solution was centrifuged twice for 1 h at 30,000 rcf at 20 °C (Centrifuge 5430 R, Eppendorf). About ¾ of the supernatant was collected and pellets were discarded to remove unsuspended material. The supernatants were filtered through centrifugal filter units (100 kDa MWCO, Amicon Ultracel, Merck Millipore Ltd.), washed with water four times to remove small molecules and excess polymer. A litmus paper test was performed to confirm the removal of acid/base from the suspension. All the aqueous suspensions were prepared using ultrapure water (18.2 mΩ). The resulting dark black suspensions of polymer-nanotube complexes were stored at room temperature under ambient conditions.

### Characterization of polymer-nanotube complexes in solution

The suspensions were characterized by visible-near-infrared (VIS–NIR) absorbance and NIR fluorescence spectroscopies. The VIS–NIR absorption spectra were measured with a JASCO V-670 spectrophotometer (Tokyo, Japan). The concentration of nanotubes was calculated using the extinction coefficient Abs_625_ = 0.036 L mg^−1^ cm^−1^. Near-infrared photoluminescence excitation/emission (PL) measurements were performed on a home-built instrument consisting of an IsoPlane SCT 320 spectrograph and PIoNIR InGaAs detector (Princeton Instruments) connected to an Olympus IX71 inverted microscope as reported^38^. The excitation was via using a SuperK EXTREME supercontinuum white light laser source connected to a Varia variable bandpass filter accessory capable of tuning the output from 490–825 nm with a bandwidth of 20 nm (NKT Photonics). The excitation beam was filtered with a long pass dichroic mirror with a cut on wavelength of 900 nm. The excitation light was shaped and fed into the back of the microscope and passed through a 20X nIR objective (Olympus) to illuminate 100 μL aqueous nanotube sample at a concentration of 2–5 mg/L in 96-well plates. The emission was collected through the ×20 near-infrared objective (Olympus), passed through a dichroic mirror and recorded from 915 to 1354 nm with a resolution of ~0.7 nm. The light was collected by a NIRvana InGaAs 640 × 512 pixel array detector (Princeton Instruments). A HL-3-CAL-EXT halogen calibration light source (Ocean Optics) was used to correct for wavelength-dependent artifacts, according to the manufacturer’s specification. A Hg/Ne pencil style calibration lamp (Newport) was used to calibrate the spectrometer wavelength. Ensemble spectroscopy measurements were performed using the aforementioned spectrometer upon excitation at 730 nm using the supercontinuum laser source.

In brief, data were collected using a custom Labview (National Instruments) automation program. The NIR spectra were corrected for: (i) wavelength-dependent detector efficiency, using an Ocean Optics reference light source, according to the manufacturer’s specification, and (ii) pixel to wavelength calibration, using a HgNe pen lamp and an internal calibration routine provided by Princeton Instruments. The calibrated data were first processed by subtracting the blank signal obtained from a well containing buffer only. In the background-subtracted data, an automated fitting routine (written in Matlab (R2014a, The MathWorks), code available upon request) was used to identify emission peaks, and to fit each emission spectrum with a Lorentzian function. The center wavelength, peak height and linewidth were obtained from the fitted spectra. Graphs were plotted using GraphPadPrism (7.01) software. The reference center wavelength in solution was within ±0.23 nm between measurements. Reference data were collected separately for each experiment.

Note: For photoluminescence excitation/emission (PLE) maps, areas of much low intensity for emission at 1060 nm and excitation around 575, 740, and 800 nm, were sometimes noted, unlike in PLE maps of similar carbon nanotubes, (Supplementary Fig. 2) due to over-subtracting the blank from the data. In our instrument, which incorporates a fluorescence microscope, the excitation results in emission from trace neodymium present in the optical elements within the microscope objective itself (Olympus LCPlan N 20 × 0.45 IR) at ~1060 nm, due to excitation peaks (at ~575, 740 and 800 nm). In some SWCNT solutions, removal of this strong background emission can result in over-subtraction, resulting in the zero (dark blue) regions (Supplementary Fig. 2).

Zeta potential (surface charge) measurements were conducted by suspending polymer-nanotube complexes (~1–2 mg/L) in ultrapure water (18.2 mΩ) in a 1 mL folded capillary cell (Malvern). Measurements were conducted at room temperature using a Zetasizer Nano-ZS instrument (Malvern).

For atomic force microscopy, carboxy-PCD-SWCNTs were treated with albumin (50 mg/L) and incubated at room temperature for 30 min; excess albumin was removed via centrifugal filtration (100 kD MWCO filter, Amicon; 10,000 rcf, 6 min) and reconstituted in water. The polymer-nanotube complexes (1 mg/L) were deposited onto a freshly cleaved mica surface (Pelco Mica Disc, V1, Ted Pella) and allowed to stand for 1 min before washing the surface with deionized water two times to remove unbound carbon nanotubes. The mica surface was dried at room temperature with ultrapure nitrogen prior to imaging. AFM images were collected using Asylum MFD-3D-BIO in AC mode using AC240TS tips (Asylum Research). The typical scan size was 2 µm and scan rate was 0.25 Hz–0.5 Hz. The images were processed with Igor software.

### Sensor response to albumin in solution

Human serum albumin (≥99% pure, essentially globulin free lyophilized powder, Sigma–Aldrich) solution was prepared by dissolving albumin in sterile PBS (1×) at room temperature. The sample solution was either used immediately or stored at 4 °C and used within a week. An aqueous suspension of polymer-SWCNTs complexes (90 µL per well) was deposited into a 96-well plate. Albumin (specified concentration, 10 µL per well) was added to the well and the solution was thoroughly mixed using a pipette. The solution was then allowed to equilibrate at room temperature for 1 h, except in the kinetic measurements experiments, prior to optical measurements. The samples were excited at 730 nm. All the measurements were performed in triplicate and repeated at least three times. The data presented are from three technical replicates for the experiments. The results were found to be reproducible within 5–10% error.

Kinetic measurements of the nanotube response to albumin in solution was performed following a similar protocol as described above. All the measurements were performed under ambient conditions. Briefly, the polymer-nanotube complexes in solution (90 µL, 2 mg/L) were added to a 96-well plate and excited at 730 nm; the emission was continuously measured. After 10 min, albumin solution (10 µL) was quickly added to obtain a final concentration of albumin of 100 mg/L, mixed thoroughly by using a pipette. Emission was collected over 60 min with a 1 s duration of acquisition per spectrum.

### Paint-immobilized nanosensor preparation

Carbon nanotube paint composites were prepared by blending together an aqueous suspension of Carboxy-PCD-SWCNT complexes (200 μL, 7 mg/L concentration) and artist acrylic gel medium (~2 g Matte Super Heavy Acrylic Gel form Liquitex^®^). The paint mixture was applied onto a glass surface to make a thin film and allowed to dry at room temperature overnight (~10 h). The painted surface was ringed with DI water twice to remove unbound nanotubes followed by further drying under ambient conditions for 6 h. A blank paint film without nanotubes was prepared and used for background spectroscopic measurements.

### Fluorescence spectroscopy on paint-immobilized nanosensor

Paint-immobilized polymer-nanotube complexes were measured using a custom-built reflectance probe-based near-infrared spectroscopy system described below. The paint, bound to a glass surface, was measured upon 730 nm excitation through the fiber optic probe. Near-infrared emission was collected from the same fiber bundle and collected via spectrometer-coupled InGaAs array detector. 50 μL of albumin solution (Microalbumin calibrators, Abbott Laboratories, USA) of specified concentration was deposited on the paint surface, and emission was collected again after 1 h incubation at room temperature. The fluorescence collected in the same area of the paint prior to albumin treatment was used as the control. To measure responses from clinical microalbuminuria samples using nanosensor paint, the urine samples were first processed minimally. Urine samples were filtered using 50kD MWCO filters and the flow through was discarded. The residue was reconstituted with the same volume of DI water. The sample (50 μL) was applied to the paint surface and nIR fluorescence was measured. For control experiment, healthy urine sample was used.

The optical measurement system consisted of a continuous wave 1 watt 730 nm diode laser (Frankfurt) connected to a bifurcated fiber optic reflection probe bundle (Thorlabs) and Czerny-Turner spectrograph with 303 mm focal length (Shamrock 303i, Andor). The sample leg of the bundle included one 200 µm, 0.22 NA fiber optic cable for sample excitation located in the center of six 200 µm, 0.22 NA fiber optic cables for collection of the emitted light. Long pass filters were used to filter emission <1050 nm. The emission light was focused through a 410 μm slit into the spectrograph. The light was dispersed using a 85 g/mm grating with 1350 nm blaze wavelength and collected with an iDus InGaAs camera (Andor). Spectra were fit to Voigt functions using custom Matlab code.

### Nanosensor response in microalbuminuria urine samples

Waste human urine specimens collected with IRB approval were used at MSKCC. The samples were stored at 4 °C for 4–6 days when needed. The urine samples were centrifuged (1000 rcf, 5 min) and the supernatant was collected. The supernatant was used directly or filtered through 50 kD MWCO filters twice, the flow through was discarded and the residue was reconstituted in DI water. Nanosensor solution (10 μL) was added to urine sample (90 μL), thoroughly mixed, incubated at room temperature for specified time (1–4 h) and the nIR fluorescence was measured. The final concentration of nanotubes in aqueous suspension was 2–5 mg/L.

Albumin degradation, to simulate conditions in urine, was performed by following a literature protocol (Kania et al. Urinary proteases degrade albumin: implications for measurement of albuminuria in stored samples. Ann Clin Biochem 47, 151–157, 2010). Briefly, urine sample was spiked with albumin and pH was adjusted by adding dilute hydrochloric acid until the desired pH was reached. The mixture was then incubated at room temperature for 30 min, followed by neutralization by addition of sodium bicarbonate. Degradation was confirmed by SDS-PAGE gel. The lower pH caused slight quenching but no significant effect on emission wavelength (Fig. 2d, e).

### Reporting summary

Further information on research design is available in the Nature Research Reporting Summary linked to this article.

## Supplementary information

Supplementary Information

Reporting Summary

## Data Availability

Data supporting the findings of this study are available within the paper and its Supplementary Information files. All other relevant data are available from authors upon reasonable request.

## Code availability

The custom MATLAB code is available from the corresponding author upon request.
