# Supplementary material for: Synthetic molecular recognition nanosensor paint for microalbuminuria
Source: Nat Commun. 2019 Aug 9;10:3605. doi: 10.1038/s41467-019-11583-1 (PMC6689023; doi:10.1038/s41467-019-11583-1)
Supplement: Supplementary file 1 — Supplementary Information [file 41467_2019_11583_MOESM1_ESM.pdf]

Supplementary Information for:

Synthetic Molecular Recognition Nanosensor Paint for Microalbuminuria

Januka Budhathoki-Uprety[1,2], Janki Shah[1], Joshua A. Korsen[1, 3], Alysandria E. Wayne[1,4], Thomas V. Galassi[1,3], Joseph R. Cohen[1], Jackson D. Harvey[1,3], Prakrit V. Jena[1], Lakshmi V. Ramanathan[1], Edgar A. Jaimes[1,3] and Daniel A. Heller[1,3]*

[1]Memorial Sloan Kettering Cancer Center, New York, New York 10065, United States

[2]Department of Textile Engineering, Chemistry, and Science, North Carolina State University, Raleigh, NC 27695, United State

[3]Weill Cornell Medical College, New York, New York 10065, United States [4]Washington University in St. Louis, St. Louis, MO 63130, United States

*Correspondence to: hellerd@mskcc.org

Supplementary Note 1

Synthesis and characterization of polycarbodiimide (PCD) polymers

Chemicals

Reagents and solvents were purchased from Sigma-Aldrich, Milwaukee, WI, Acros Organics, and Fisher Scientific, Fair Lawn, NJ, and used as received. Anhydrous toluene and anhydrous and inhibitor-free diethyl ether were used in catalyst synthesis and purification. Anhydrous and inhibitor-free tetrahydrofuran was used for 'click chemistry'.

Material characterization

Materials were synthesized and characterized as reported previously[1-3]. NMR data were recorded on a Bruker Advance III Ultrashield Plus 500 MHz spectrometer at room temperature. The chemical shift values were reported relative to TMS (δ = 0.00 ppm) as an internal standard. Fourier transform infrared (FTIR) spectra were acquired using a Bruker Optics Tensor 27 FTIR spectrometer using NaCl disc. Wavenumbers in cm^{-1} are reported for characteristic peaks. High resolution mass spectra (HRMS) were obtained on a Waters LCT-Premier XE mass spectrometer by electrospray ionization. Size exclusion chromatography (SEC) was performed on a Viscotek GPCmax system (Malvern Instruments) equipped with ViscoGEL columns (I-MBMMW-3078 and I-MBLMW-3078 in series) connected to a Viscotek TDA 305 triple detector array at 30 °C using THF as an eluent to determine relative molecular weights of the polymers. Polystyrene standards were used for the calibration of the instrument. Polymer samples were dissolved in THF, and the solutions were filtered through 0.45 μm PTFE filters prior to injection. The flow rate was 1.0 mL/min, and injector volume was 100 μL. OmniSEC software was used to calculate the molecular weight.

Synthesis of catalyst, *R*-BINOL-titanium(IV)-diisopropoxide[4]:

All manipulations were performed inside a glove box under an argon atmosphere. All the accessories (glassware, magnetic stir bar and spatula) were cleaned thoroughly and dried overnight in an oven. Anhydrous solvents (≥99.8%) were purchased from the vendors and used as received. The glassware and chemicals were placed in the glovebox antechamber for 5 minutes to remove air prior to use. Inside glove box, (*R*)-BINOL (6.89 mmol, 2g) was mixed with toluene (5 mL) in a 20 mL glass scintillation vial. Titanium tetraisopropoxide (6.98 mmol, 1.98 g) diluted in toluene (5 mL) was added slowly to stirring suspension of (*R*)-BINOL. The solid dissolved within minutes forming a dark red solution. The reaction mixture was then transferred to a 100 mL Schlenk tube equipped with a magnetic stir bar, the tube was

capped, and removed from the glove box. The reaction mixture was stirred overnight at room temperature and the solvent was removed by vacuum distillation. Dark red solid thus formed was dissolved in diethyl ether (10 mL) that *via* cannula transfer. The compound was recrystallized at -20 °C from diethyl ether. The excess solvent was removed *via* cannula transfer. The recrystallization process was repeated two more times, the compound was dried under reduced pressure for 24 h and stored at -30 °C under inert atmosphere (inside glove box).

Synthesis of polymer:

Urea derivative (**1**), monomer (**2**), and corresponding alkyne polycarbodiimide (PCD) polymer were prepared following a previously reported procedure[1] and briefly described below.

Urea derivative, *1-phenyl-3-(prop-2-yn-1-yl)urea*, **1**. Propargyl amine (1.20 g, 21.78 mmol, 1.1 equiv) was diluted in anhydrous dichloromethane (30 mL) and added to phenylisocyanate (2.36 g, 19.80 mmol, 1.0 mol equiv) in dichloromethane (30 mL), stirred at low temperature, and kept cold in an ice bath. The reaction mixture was then allowed to warm to room temperature. A white precipitate resulted shortly after mixing the amine with phenylisocyanate. The reaction mixture was stirred for 3 hours at room temperature. The white solid was filtered and further purified by recrystallization in dichloromethane at 4°C to obtain white crystalline solid **1,** 92% yield. **^{1}H NMR** (500 MHz, DMSO-d6, δ ppm): reference DMSO-d6 = 2.50 ppm, δ = 8.56 (s, 1H), 7.40 (d, *J* = 7.65 Hz, 2H), 7.23 (t, *J* = 7.60 Hz, 2H), 6.91 (t, *J* = 7.35 Hz, 1H), 6.45 (t, *J* = 5.60 Hz, 1H), 3.90 (dd, *J* = 5.70 Hz, 2.45 Hz, 2H), 3.09 (t, *J* = 2.45 Hz, 1H). **^{13}C NMR** (125 MHz, DMSO-d6, δ ppm): reference DMSO-d6 = 39.51 ppm, δ = 154.7, 140.1, 128.6, 121.4, 117.8, 82.1, 72.9, 28.7. **HRMS (ESI)** $[M+H]^+$ m/z calcd for $C_{10}H_{11}N_2O$, 175.0871; found, 175.0863.

Monomer, *N-((prop-2-yn-1-ylimino)methylene)aniline*, **2**.Triethyl amine (2.07 g, 20.51 mmol, 2.5 equiv) was added to a suspension of dibromotriphenylphosphorane (4.15 g, 9.84 mmol, 1.2 mol equiv) in dichloromethane (2 mL) and stirred at low temperature under inert atmosphere. After stirring the mixture for 5 minutes, compound **1** (1.66 g, 8.20 mmol, 1.0 equiv) was added in three equal portions at an interval of 5 minutes and the reaction mixture was stirred at low temperature until reaction completion. The dehydration of the urea derivative into carbodiimide monomer was monitored by the formation of a very strong FTIR signal at ~ 2120-2140 cm^{-1}. Upon completion of the reaction, hexane (30 mL) was added to precipitate side products. The monomer compound was then extracted from the solid by hexanes (~20 mL X 4). The fractions were combined and the solvent was removed using a rotary evaporator. The crude

monomer was further purified by column chromatography on silica gel using ethyl acetate:hexanes (1:2) and dried under reduced pressure to obtain **2** as a colorless oil.85% yield.

^{1}H NMR (500 MHz, $CDCl_3$, δ ppm): reference TMS = 0 ppm, δ = 7.31-7.28 (m, 2H), 7.16-7.14 (m, 3H), 4.08 (d, *J* = 2.45 Hz, 2H), 2.44 (t, *J* = 2.50 Hz, 1H). **^{13}C NMR** (125 MHz, $CDCl_3$, δ ppm): reference TMS = 0 ppm, δ = 139.5, 139.0, 129.4, 125.5, 124.0, 79.0, 73.5, 36.0. **FTIR** (thin film, cm^{-1}): characteristic absorption from terminal alkyne group and monomer; 3302 (terminal alkyne), 2119 (vs, carbodiimide). **HRMS (ESI)** $[M+H]^+$ m/z calcd for $C_{10}H_9N_2$, 157.0766; found, 157.0761.

Alkyne polycarbodiimide (PCD) polymer. All manipulations for polymerization were performed at room temperature inside an MBraun UNIlab drybox under inert atmosphere. Briefly, the catalyst, *R*BINOL-titanium(IV)-diisopropoxide[4], (60 mg,0.12 mmol,) was added to the stirring monomer **2** (678 mg, 4.34 mmol) at room temperature. The mixture turned to a dark viscous liquid and solidified within an hour. After 24 hours, the orange-red solid was dissolved in chloroform (3 mL) and precipitated into methanol-hexane mixture. Light yellow solid was separated, re-dissolved in chloroform, re-precipitated, and dried to obtain **alkyne PCD** as light yellow solid (90% yield). FTIR (thin film, cm^{-1}): characteristic absorption from terminal alkyne group and polymer backbone; 3304 (terminal alkyne C–H), 2123 (alkyne triple bond, C≡C), 1631 (imine in polymer backbone, C=N). **Mn** = 36, 608, PDI = 1.35. **^{1}H NMR** (500 MHz, $CDCl_3$, δ ppm): reference TMS = 0 ppm, δ = 7.28–6.84 (br), 5.35–5.29 (br), 4.20 (br), 3.14 (br), 2.07–0.75 (br).

2-azidoacetic acid (3) was synthesized and characterized following literature procedures.[5] **^{1}H NMR** (500 MHz, $CDCl_3$, δ ppm): reference TMS = 0 ppm, δ = 10.16 (s, 1H), 3.98 (s, 2H). **^{13}C NMR** (125 MHz, $CDCl_3$, δ ppm): reference $CDCl_3$ = 77.36 ppm, δ = 174.6, 50.3. **FTIR** (thin film, cm^{-1}): characteristic absorption from the azide group; 2109 (s). **HRMS (ESI)** $[M-H]^-$ m/z calcd for $C_2H_2N_3O_2$, 100.0147; found, 100.0147.

'Click' chemistry' on alkyne PCD Polymer. Alkyne PCD (20 mg) was dissolved in tetrahydrofuran (4 mL, anhydrous and inhibitor-free). To the stirring polymer solution under inert atmosphere, azide compound (1.2 mol equiv per alkyne unit), triethyl amine (0.1 mL) and CuI (10 mol %) were added. The reaction mixture was stirred overnight under an argon atmosphere. Coupling of small molecules azides to alkyne side chains in polymers was monitored by FTIR analysis. Upon completion of the reaction, the solvent was removed using a rotary evaporator. The polymer was washed with diethyl ether, separated by filtration and dried under reduced pressure. FTIR analysis of final polymers showed full conversion of all

alkyne repeat units in click reaction. Limited solubility of final polymers posed difficulty in GPC measurements and NMR analysis.

Supplementary Figures

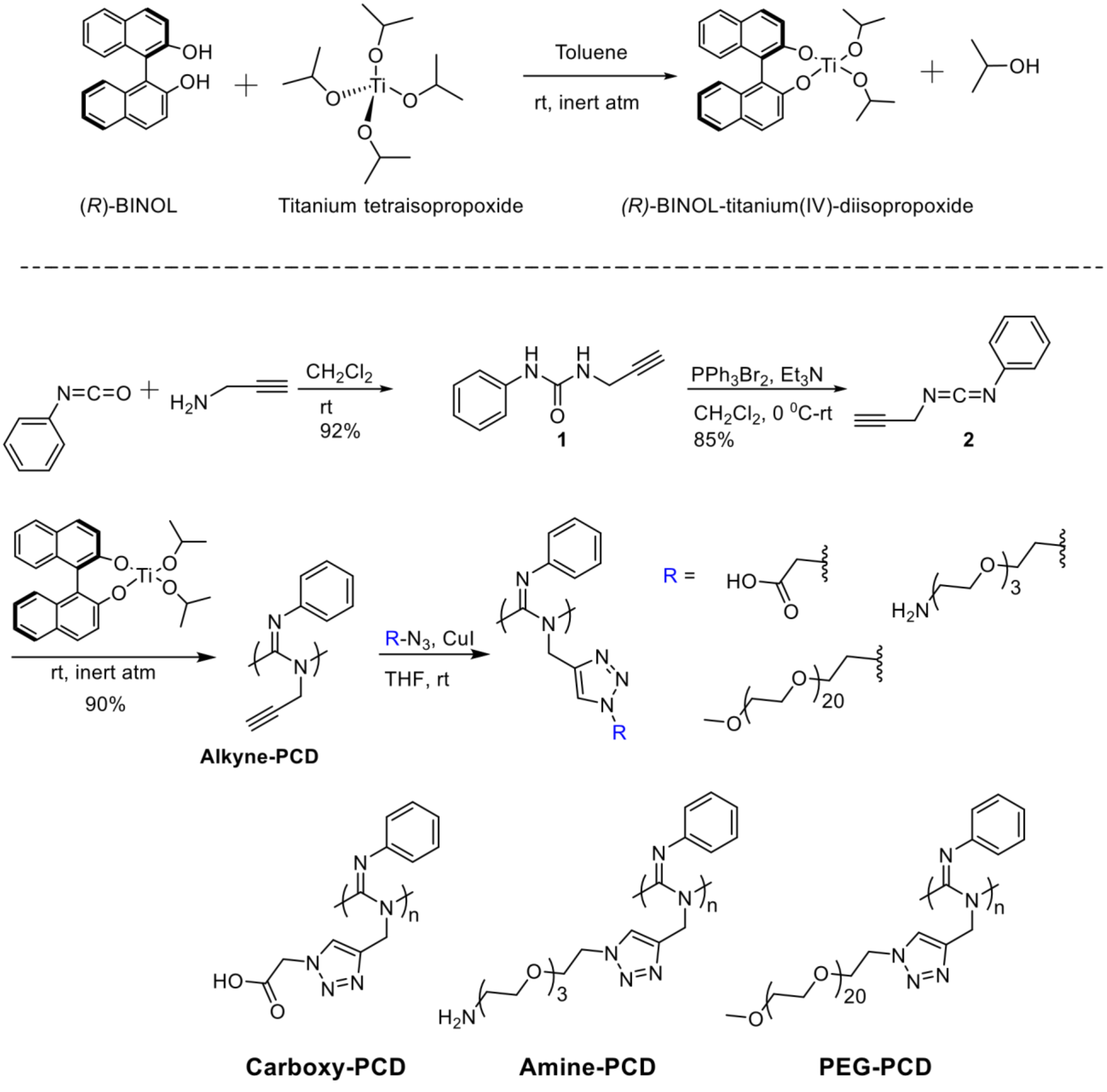

Supplementary Figure 1. Synthesis of polycarbodiimide polymers.

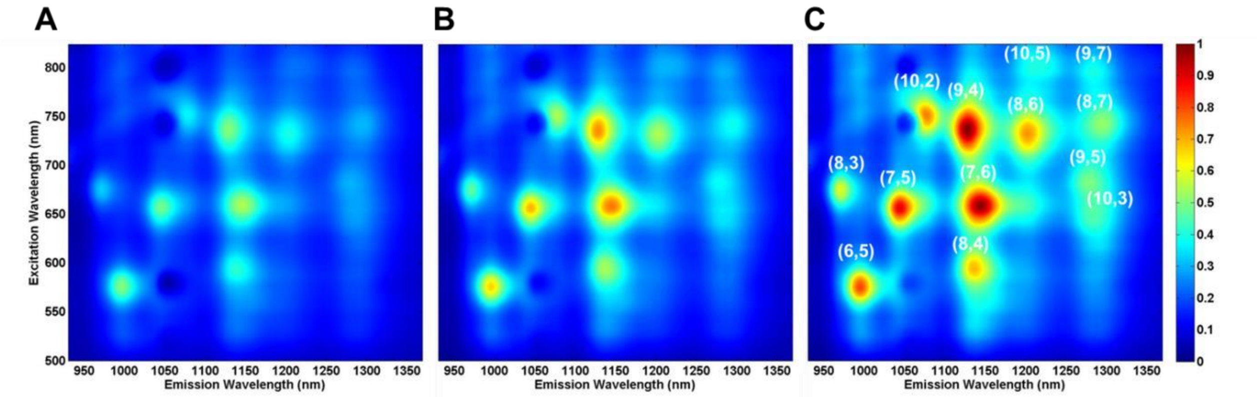

Supplementary Figure 2. Photoluminescence excitation/emission plot of Carboxy-PCD-SWCNTs without albumin (A) and upon addition of 25 mg/L (B) and 100 mg/L albumin (C). (n,m) represents chiralities of nanotube species. Nanotube emission intensity was normalized globally.

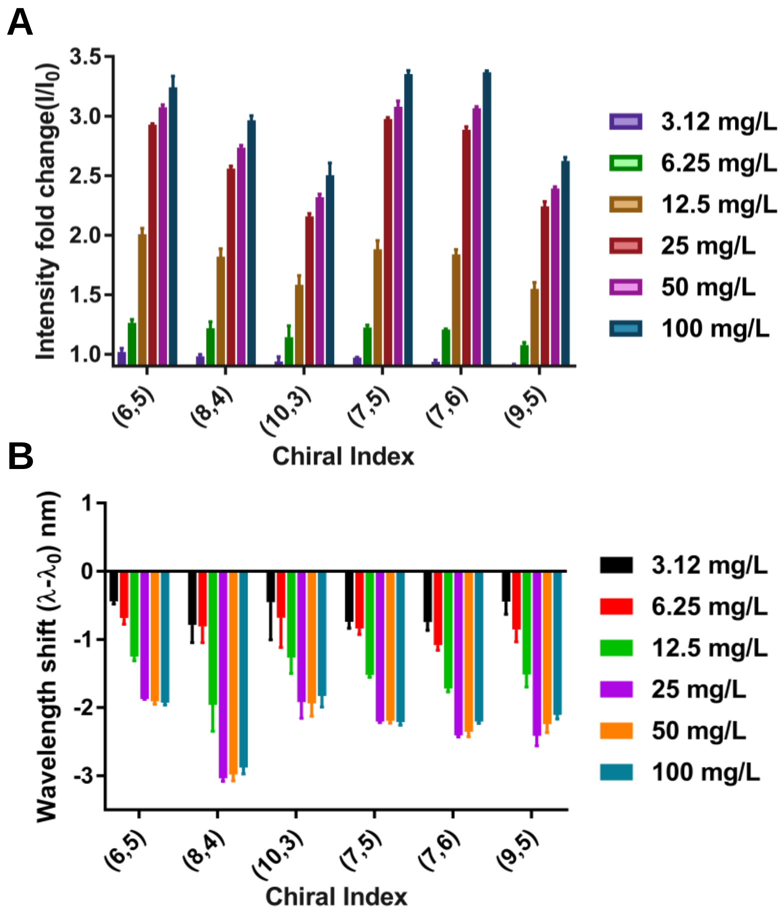

Supplementary Figure 3. Response of carboxy-PCD-SWCNTs upon addition of albumin. (A) The intensity responses of several different carbon nanotube chiralities to albumin, in PBS. (B) The emission

wavelength response of several different carbon nanotube chiralities with albumin dose response. The responses were compared to the buffer control.

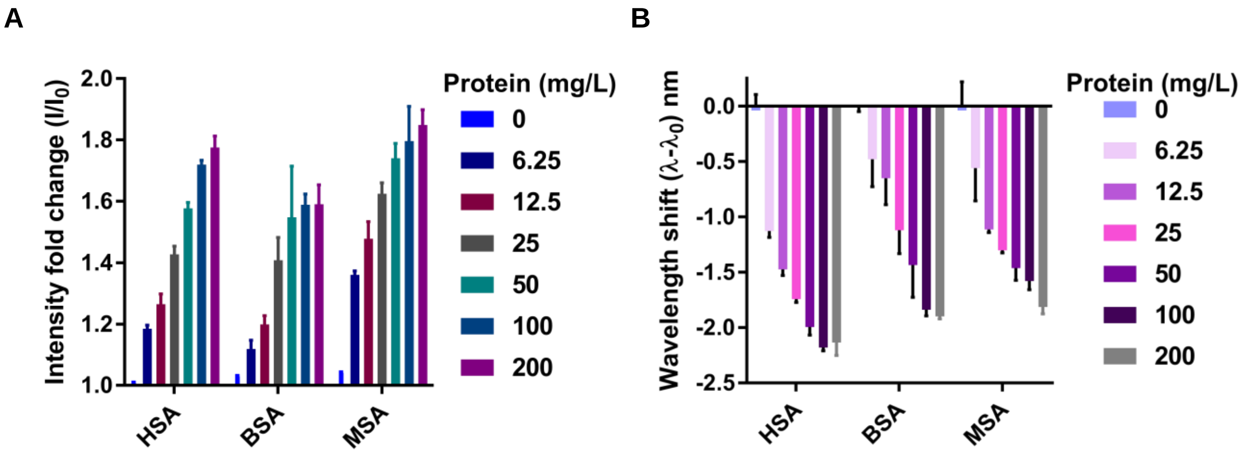

Supplementary Figure 4. Nanosensor response for proteins with conserved sequences, albumin from bovine, human and mouse. A) Intensity responses of (9,4) nanotubes to serum albumin from various sources, human serum albumin (HSA), Bovine serum albumin (BSA) and mouse serum albumin (MSA). B) Wavelength responses of (9,4) nanotubes to human serum albumin (HSA), Bovine serum albumin (BSA) and mouse serum albumin (MSA). The responses were compared to the buffer control.

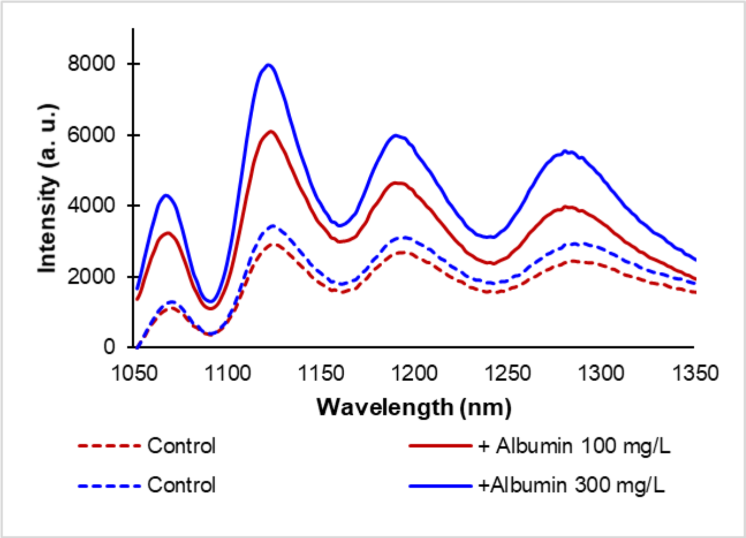

Supplementary Figure 5. Photoluminescence emission spectra of carboxy-PCD-SWCNT complexes immobilized as a paint, before and upon addition of albumin (100 mg/L and 300 mg/L). Solid lines denote spectra from albumin-treated samples, whereas dotted lines denote those without albumin treatment. Data were obtained from similar areas of the paint before and after treatment.

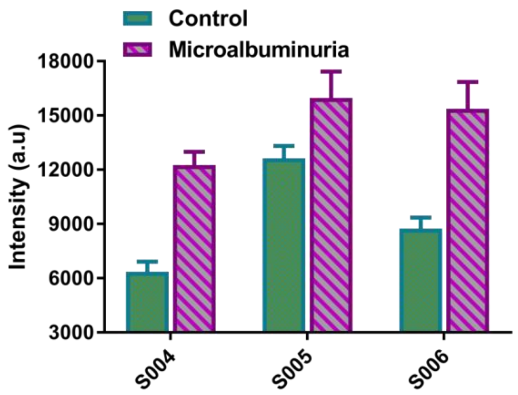

Supplementary Figure 6. Emission intensity response of the paint-immobilized nanosensor to clinical urine samples from patients with microalbuminuria, after filtration using a 50 kD MWCO filter. Controls denote the same regions of the paint material before treatment with each clinical sample (S004, S005, and S006).

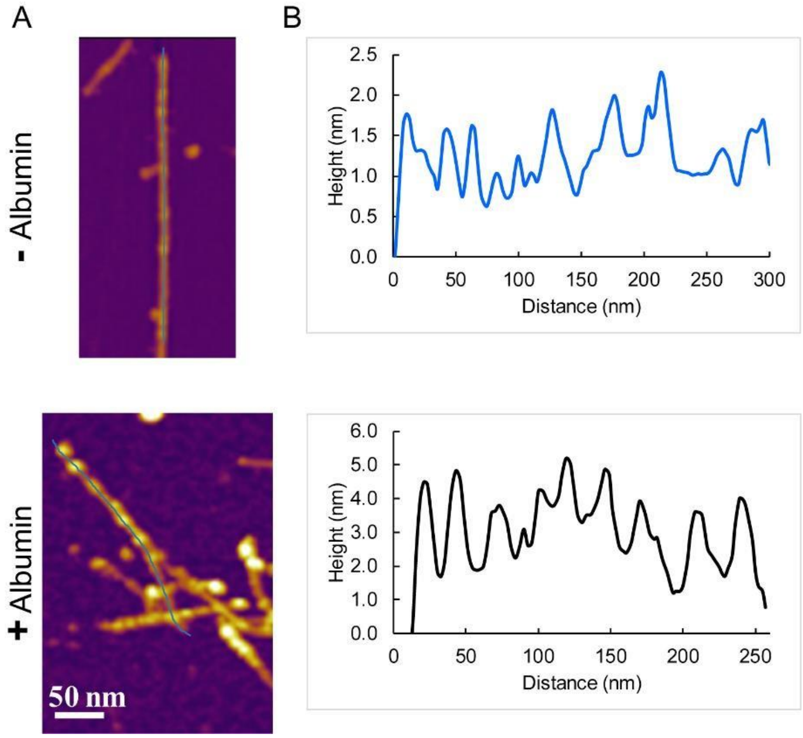

Supplementary Figure 7. Atomic force microscopy (AFM) measurements of carboxy-PCD-SWCNT complexes in the absence and presence of albumin. A, AFM height images of carboxy-PCD-SWCNTs without albumin (top) and after exposure to albumin (bottom). B) Height profiles acquired from the image without albumin (top) and after exposure to albumin (bottom).

Supplementary Tables

Supplementary Table 1: Methods for albumin measurements in urine[9].

Precipitation	Sulphosalicylic acid, Boiling
Dye-binding	Biuret, tetrabromophenol, albumin blue 580
Immune-assays	Nephelometry, turbidimetry, radioimmunochemistry
Molecular size	HPLC

Supplementary Table 2. Albumin and total protein measured via turbidimetry methods. Samples (MA1MA4) were then diluted to approximately 100 mg/L albumin concentration prior to measurements using nanosensor.

Sample	Albumin (mg/L)	Total protein (mg/L)	Alb/Total protein
MA1	100	167.04	0.5986
MA2	100	189.05	0.5289
MA3	100	207.51	0.4819
MA4	100	229.26	0.4361

Supplementary Table 3. Albumin concentration in microalbuminuria clinical samples.

Sample	Albumin (mg/L)
S004	131.1
S005	168.2
S006	127.7

Supplementary References

1. Budhathoki-Uprety, J. & Novak, B. Synthesis of Alkyne-Functionalized Helical Polycarbodiimides and their Ligation to Small Molecules using 'Click' and Sonogashira Reactions. *Macromolecules* **44**, 5947-5954 (2011).
2. Budhathoki-Uprety, J., Jena, P.V., Roxbury, D. & Heller, D.A. Helical Polycarbodiimide Cloaking of Carbon Nanotubes Enables Inter-Nanotube Exciton Energy Transfer Modulation. *J Am Chem Soc* **136**, 15545-15550 (2014).

3. Budhathoki-Uprety, J. et al. Polymer cloaking modulates the carbon nanotube protein corona and delivery into cancer cells. *J Mater Chem B* **5**, 6637-6644 (2017).
4. Tang, H., Boyle, P. & Novak, B. Chiroptical switching polyguanidine synthesized by helix-senseselective polymerization using [(R)-3,3 '-dibromo-2,2 '-binaphthoxy](di-tert-butoxy)titanium(IV) catalyst. *Journal of the American Chemical Society*, 2136-2142 (2005).
5. Srinivasan, R. et al. High-throughput synthesis of azide libraries suitable for direct "click" chemistry and in situ screening. *Org Biomol Chem* **7**, 1821-1828 (2009).
